# Androgen-Induced Lactic Acid Accumulation Contributes to the Apoptosis of Ovarian Granulosa Cells in Polycystic Ovary Syndrome Mice

**DOI:** 10.3390/antiox14101235

**Published:** 2025-10-14

**Authors:** Bining Zhao, Liting Fan, Mengfei Liu, Haowen Wu, Youyou Zhang, Qiyang Shen, Jihong Kang

**Affiliations:** 1 Department of Physiology and Pathophysiology, School of Basic Medical Sciences, State Key Laboratory of Vascular Homeostasis and Remodeling, Peking University, Beijing 100191, China; xiyings@163.com (B.Z.); lmf01282025@163.com (M.L.); haowen_wu@yeah.net (H.W.); zhanguu@bjmu.edu.cn (Y.Z.); shenqiyang2018@sina.com (Q.S.); 2 School of Basic Medicine, Shanxi Medical University, No.56, Xinjian South Road, Yingze District, Taiyuan 030001, China; fanliting@sxmu.edu.cn

**Keywords:** polycystic ovary syndrome (PCOS), hyperandrogenemia, lactic acid, pH value, mitochondria, apoptosis

## Abstract

Background: Polycystic ovary syndrome (PCOS) is the leading cause of anovulatory infertility. The apoptosis of granulosa cells (GCs) is strongly associated with the impaired follicular development in PCOS. The underlying mechanisms, however, remain incompletely elucidated. A significant increase in circulating lactic acid, an anaerobic respiration product, has been detected in PCOS patients. Yet, alterations in local ovarian lactic acid levels and their impact on GCs remain unknown. Methods: PCOS mouse models were established via 20-day daily subcutaneous dehydroepiandrosterone (DHEA) injections. In vitro experiments utilized DHEA-treated KGN cells to mimic hyperandrogenic conditions. Circulating, ovarian, and cellular lactic acid concentrations were quantified. Intracellular and extracellular pH values were measured using BCECF-AM fluorescent probe and a blood gas analyzer, respectively. Apoptosis was assessed through both flow cytometry and TUNEL assay. The antioxidant N-acetylcysteine (NAC) was used to investigate its effects on lactic acid levels and the subsequent GC apoptosis. Results: High androgen levels caused mitochondrial damage, promoted anaerobic glycolysis and led to lactic acid accumulation, inducing decreased intracellular pH and thus apoptosis of GCs. The antioxidant NAC effectively alleviated oxidative stress, mitigated mitochondrial damage, and decreased lactic acid levels and apoptosis in KGN cells. In PCOS mice, NAC improved ovarian morphology, but it did not affect the estrous cycle of the mice. Conclusions: Hyperandrogenemia-induced mitochondrial dysfunction caused the accumulation of lactic acid and thus apoptosis of ovarian GCs in PCOS mice. NAC enhanced mitochondrial function, consequently decreasing lactic acid concentrations. These findings suggest novel therapeutic targets for PCOS.

## 1. Introduction

Polycystic ovary syndrome (PCOS) affects 11–13% of reproductive-aged women and causes a substantial economic burden on the nation [1]. The key reproductive features of this syndrome include hyperandrogenism, ovulatory dysfunction and polycystic ovaries with multiple small antral follicles and/or enlarged ovaries [2]. Moreover, PCOS is associated with metabolic comorbidities, including obesity, insulin resistance (IR)/hyperinsulinemia, type 2 diabetes (T2D) and hyperlipidemia.

PCOS is the most common cause of anovulatory infertility in individuals of reproductive age, with granulosa cell dysfunction constituting a fundamental pathological component. Numerous clinical [3,4] and experimental studies [5] have demonstrated a pivotal role of granulosa cell (GC) apoptosis in the pathogenesis of PCOS, wherein dysfunctional GCs impair folliculogenesis and promote follicular atresia at the preantral stage. Results from rodent studies on the etiology of PCOS have associated the polycystic ovarian morphology with the loss of GCs. In the pathogenesis of PCOS, multiple factors contribute to the apoptosis of GCs, including hyperandrogenism, abnormal metabolic byproducts [6,7,8], exosomes [9,10,11], etc. Among these, hyperandrogenism emerges as the predominant inducer of GC apoptosis. Elevated androgens induce GC apoptosis by binding to androgen receptors (ARs), followed by activation of the downstream pro-apoptotic signaling pathways [12]. Additionally, hyperandrogenemia in PCOS triggers ovarian inflammatory responses, creating a pro-inflammatory microenvironment that exacerbates GC apoptosis via endoplasmic reticulum (ER) stress [13,14].

In our previous study, data from in vitro experiments demonstrated that apoptosis of GCs was closely associated with mitochondrial damage [14]. Notably, antioxidant treatment effectively ameliorated androgen-induced mitochondrial damage, consequently improving the metabolic phenotype of PCOS in both in vivo and in vitro models [15]. PCOS patients exhibit a significant imbalance between oxidative and antioxidant biomarkers. Importantly, those with a hyperandrogenic (HA) phenotype typically exhibit significantly elevated oxidative stress, a finding that aligns with our observations. Current evidence indicates that antioxidant compounds, such as soy isoflavones, coenzyme Q10, and astaxanthin, can ameliorate oxidative stress and mitigate endocrine disturbances in PCOS [16]. Nevertheless, their therapeutic efficacy and underlying mechanisms in ovarian function remain poorly characterized.

In our previous in vitro experiments, we observed that GCs exposed to high androgen concentrations exhibited significantly lower extracellular pH values compared with those in the androgen-free control group. Metabolomic studies by others have identified elevated circulating lactic acid levels as a discriminative metabolic feature in PCOS patients across independent cohorts [17,18]. Therefore, the current study aimed to investigate the impact of lactic acid on GCs in PCOS. PCOS mice were induced by injection of dehydroepiandrosterone (DHEA) to assay the lactic acid levels in vivo. Meanwhile, in vitro experiments were performed using the human KGN granulosa cell line treated with high androgen levels to investigate the mechanistic link between mitochondrial dysfunction, lactic acid accumulation, and the subsequent GC apoptosis.

## 2. Materials and Methods

### 2.1. Animal Treatments

Female C57BL/6J mice (aged 21 days) were purchased from the Animal Facility of Peking University Health Science Center. All animals were acclimated to standard laboratory conditions (22 ± 2 °C, 12L:12D cycle) with free access to rodent food and water. At postnatal day 25, the mice of comparable weights were randomly divided into different groups. The experiment was divided into two parts. Part 1: 12 mice of comparable weights were randomly divided into two groups. Group 1: ctrl group. The mice were subcutaneously injected daily with sesame oil (1 mL per kg body weight). Group 2: DHEA group. The mice were subcutaneously injected daily with DHEA (60 mg per kg body weight) dissolved in sesame oil, *n* = 6 per group. Part 2: 21 mice of comparable weights were randomly divided into three groups. Group 1: ctrl group. The mice were subcutaneously injected daily with sesame oil and treated with saline solution once daily by oral gavage. Group 2: DHEA group. The mice were subcutaneously injected daily with DHEA and treated with saline solution once daily by oral gavage. Group 3: DHEA + NAC group. The mice were subcutaneously injected daily with DHEA and received NAC (Sigma, St. Louis, MA, USA) (0.1 mg per 100 g body weight) dissolved in saline solution once daily by oral gavage, *n* = 7 per group. A total of 33 mice were used for the experiment. DHEA and sesame oil were purchased from Sigma-Aldrich, St. Louis, MA, USA. After the treatments for 20 days, the mice were anesthetized by intraperitoneal injection of a mixture containing ketamine hydrochloride (100 mg/kg) and xylazine (20 mg/kg). The ovarian tissues were collected, immediately fixed with fresh 4% paraformaldehyde for 4 h, and embedded in paraffin. Ovarian samples were then cut into sections of 8-μm thickness.

### 2.2. Estrous Cycle Determination

Vaginal secretions of mice were collected daily using a fine-tipped cotton swab moistened with saline and then stained with 0.1% methylene blue. The stage of the estrus cycle was determined based on vaginal cytology: predominantly nucleated epithelial cells indicated proestrus, predominantly keratinized squamous epithelial cells indicated estrus, coexistence of keratinized squamous epithelial cells and leukocytes indicated metestrus, and mucus mixed with leukocytes indicated diestrus.

### 2.3. Cell Culture

KGN cells, a human ovarian granulosa cell line, were purchased from Beijing Aitemeng Science and Technology Co (catalog number: BNCC-337610). Cells were cultured in Dulbecco’s Modified Eagle’s Medium (DMEM) supplemented with 10% FBS and 100 U/mL penicillin/streptomycin in a humidified 5% CO_2_ atmosphere at 37 °C. The culture medium was changed every two days. After reaching a confluence of approximately 90%, the KGN cells were treated with 50 μM DHEA for 24 h.

### 2.4. TUNEL Staining

The apoptosis of the ovarian tissues from the mice was detected by terminal deoxynucleotidyl transferase-mediated dUTP nick-end labeling (TUNEL) staining with a commercially available kit purchased from Elabscience Biotechnology (Wuhan, China) according to the manufacturer’s instructions. For cells, KGN cells were cultured in 6 cm dishes with glass bottoms (Beyotime, Shanghai, China) and treated with DHEA (0 and 50 μM, respectively) for 24 h. Then the apoptosis of KGN cells was detected by measuring fluorescence intensity.

### 2.5. Measurement of Intracellular pH (pHi)

The pHi values were measured using the pH-sensitive fluorescent probe BCECF-AM [19] (2′,7′-bis-(2-carboxyethyl)-5-carboxyfluorescein-acetoxymethyl ester, Beyotime, China). According to the manufacturer’s instructions, cells were incubated for 20 min at 37 °C with 1 μM BCECF-AM in Hank’s balanced salt solution. BCECF fluorescence was imaged using a standard fluorescence filter set (Olympus, Tokyo, Japan) [20]. The relative fluorescence intensity of BCECF-AM was used to determine pHi values. The results were normalized to the control group for quantitative analysis.

### 2.6. Determination of Extracellular pH Values (pHe)

KGN cells were cultured in serum-free DMEM with DHEA (0 and 50 μM, respectively) for 24 h in a humidified 5% CO_2_ atmosphere at 37 °C. The culture medium was collected and centrifuged. Following the treatment, culture supernatants were collected, and extracellular pH was measured using a blood gas analyzer (Radiometer, Copenhagen, Denmark) following the manufacturer’s protocol.

### 2.7. Analysis of Extracellular Acidification Rate

Extracellular acidification rate (ECAR) assay was performed as reported previously [21] with some modifications. In brief, 5 × 10^5^ cells were cultured per well in 24-well XF microplates and treated with DHEA (0 and 50 μM, respectively) for 24 h. The wells were injected sequentially with glucose (10 mM) to measure glycolysis, oligomycin (1 µM) to determine glycolytic capacity, and 2-DG (50 mM) to determine non-glycolytic acidification. The experiment was repeated 3 times. ECARs were normalized to the protein concentrations.

### 2.8. Analysis of Oxygen Consumption Rate Assay

Oxygen consumption rate (OCR) assay was performed as described above except for the injected drugs. OCRs were measured using a commercially available kit from Seahorse Bioscience (Seahorse Bioscience Inc., North Billerica, MA, USA) with the XF-24 Extracellular Flux Analyzer (Seahorse Bioscience). In brief, 5 × 10^5^ cells were seeded per well in 24-well XF microplates and treated with DHEA (0 and 50 μM, respectively) for 24 h. After different treatments, the cell culture medium was replaced with XF Assay Medium supplemented with 11 mM glucose and 2 mM glutamine. Cells were incubated in a CO_2_-free incubator at 37 °C for 1 h for temperature and pH equilibration. The baseline OCR was measured. The wells were then injected sequentially with oligomycin (1 µM) to measure the ATP-linked OCR, oxidative phosphorylation uncoupler carbonyl cyanide-4-(trifluoromethoxy) phenylhydrazone (FCCP, 0.5 µM) to determine maximal respiration, and rotenone (0.5 µM) and antimycin A (0.5 µM) to determine non-mitochondrial respiration. The experiment was repeated 3 times. OCRs were normalized to the protein concentrations.

### 2.9. Measurement of Lactic Acid Contents and the Activity of Lactic Acid Dehydrogenase (LDH)

The KGN cells were cultured in 6-well plates and treated with DHEA (0 and 50 μM, respectively) and/or NAC for 24 h. For ovaries, 20 μL of saline was added to each ovary sample. Ovaries were then punched with microsurgical scissors to release ovarian supernatant. After centrifugation at 12,000 rpm at 4 °C for 10 min, the supernatants were collected. The lactic acid concentration and the activity of lactic acid dehydrogenase (LDH) in the cells were then measured with lactic acid reagent kit and the LDH assay kit (Applygen, Beijing, China) according to the manufacturer’s instructions.

### 2.10. Detection of Cell Apoptosis by Flow Cytometry

KGN cells were harvested from 6-well plates using trypsin without EDTA and resuspended in Annexin V binding buffer containing Annexin V-FITC and propidium iodide (PI) (Elabscience, China). Samples were then incubated for 10 min at room temperature protected from light according to the manufacturer’s instructions. Flow cytometry data were acquired on Guava^®^ easyCyte^TM^ (Luminex, Austin, TX, USA). The obtained data were normalized to the protein concentrations in the same sample and presented as the fold of the Ctrl.

### 2.11. Detection of Reactive Oxygen Species (ROS) by DCFH-DA

Intracellular ROS were detected using the DCFH-DA fluorescence probe (Solarbio, Beijing, China) according to the manufacturer’s instructions. Briefly, treated KGN cells were washed with PBS and then incubated with DCFH-DA staining solution at 37 °C for 20 min. After that, the solution was removed and the cells were washed twice with PBS. The fluorescence of the cells was detected immediately using a fluorescence microscope (Olympus, Tokyo, Japan).

### 2.12. Determination of ATP Content

The determination of ATP contents of the cultured cells was performed as described previously by using a commercially available kit (Vigorous Biotechnology, Beijing, China) and a luminometer (Turner BioSystems, San Francisco, CA, USA). The results were normalized to the protein concentrations (nmol/mg protein) in the same sample and presented as the fold of the Ctrl.

### 2.13. Measurement of Mitochondrial DNA (mtDNA) Copy Number

Total DNA was isolated from KGN cells using a commercially available kit (Vazyme, Nanjing, China). Mitochondrial DNA (mtDNA, ND1) and nuclear DNA (nDNA, SLCO2B1) were amplified and quantified by real-time PCR using fluorescent SYBR Green PCR Master Mix (Vazyme, Nanjing, China) according to the manufacturer’s instructions. β-actin was used as an internal standard. The expression of the target gene was normalized to that of β-actin within the same sample using the 2^−△△Ct^ method. Each sample was measured in duplicate in each experiment. The mtDNA copy number was calculated by normalizing ND1 copies to SLCO2B1 copies in the same sample.

### 2.14. The Comet Assay

DNA damage was assessed with the Comet Assay Kit (Beyotime, Shanghai, China) according to the manufacturer’s instructions. Briefly, about 10^4^ treated cells were mixed with 0.7% low-melting-point agarose, layered onto Comet Assay Slides pre-coated with 1% normal-melting-point agarose. Then the slides loaded with cells were immersed in Lysis Buffer with DMSO for 1 h at 4 °C. The slides were placed in a horizontal electrophoresis tank containing electrophoresis buffer at room temperature for 1 h to allow DNA unwinding, followed by electrophorese at 25 V for 30 min. After washing three times with neutral buffer, 20 µL of propidium iodide solution was applied to stain the slides. Individual cells were captured under a fluorescence microscope (Olympus, Tokyo, Japan).

### 2.15. Western Blot Analysis

Western blot was performed as described previously [20]. Briefly, proteins from cells were extracted and quantified by a bicinchoninic acid (BCA) Kit (Thermo Scientific, Waltham, MA, USA). Aliquots of 10 μg total protein of each sample were separated by 8–12% SDS-PAGE and transferred to a nitrocellulose (NC) membrane. The membrane was blocked in 5% skimmed milk for 1 h at room temperature and probed with primary antibodies at 4 °C overnight. The antibodies to cytochrome c oxidase subunit 4 (COX4) and AIF were purchased from Emarbio Science & Technology and ABclonal Biotechnology (Wuhan, China), respectively. β-actin was used as an internal control of total protein and the antibody was bought from ZSGB-BIO (Beijing, China). The membrane was washed with TBST buffer and then incubated with a horseradish peroxide-conjugated secondary antibody (ZSGB-BIO, Beijing, China) at room temperature for 1 h. Blots were detected with chemiluminescence system (Tanon, Shanghai, China). Protein expression levels were quantified using Image J 10.1.2 software.

### 2.16. Statistical Analysis

Data are presented as mean ± SEM. Statistical analysis was performed using Prism software (version 8.0; GraphPad). The effects of the treatments were analyzed by unpaired Student‘s t-test for comparisons between two groups and by one-way analysis of variance (ANOVA) followed by Bonferroni’s posttest for multiple comparisons within multiple groups. *p* < 0.05 was considered statistically significant.

## 3. Result

### 3.1. High Concentrations of Androgen Reduce Extracellular pH by Increasing Lactic Acid Production in Granulosa Cells

**Figure 1:**
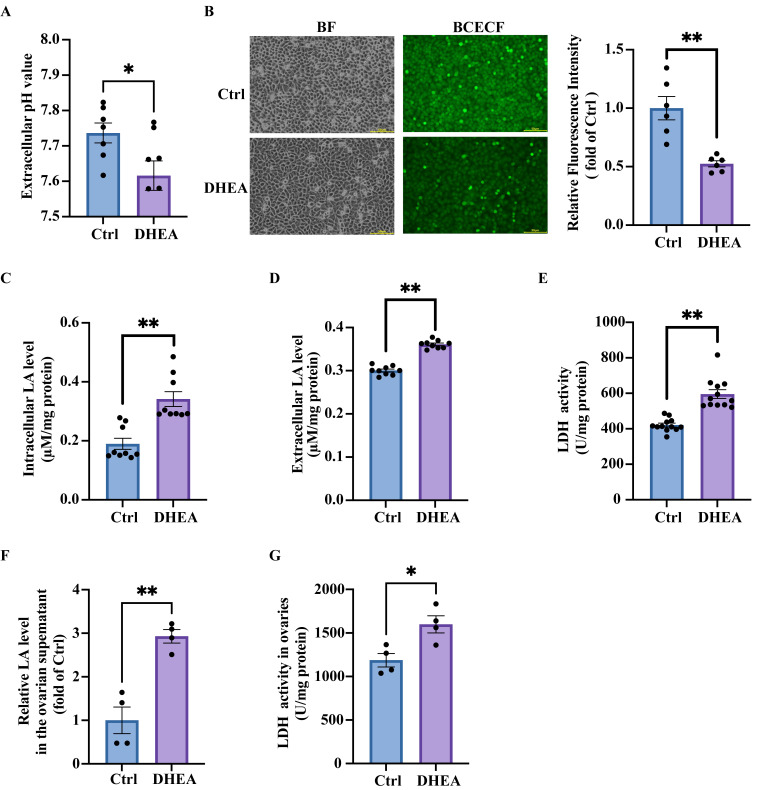
DHEA induces a decrease in pH and lactic acid accumulation in granulosa cells. KGN cells were treated with DHEA (0, 50 μM) for 24 h: (**A**) pH value of culture medium measured by a blood gas analyzer. n = 7 per group. (**B**) Representative fluorescence micrographs of intracellular pH determined by BCECF and the statistical analysis of fluorescence intensity. Scale bar = 500 μm. n = 6 per group. (**C**) The intracellular lactic acid (LA) concentrations in cells. n = 9 per group. (**D**) The lactic acid concentrations in culture medium. n = 9 per group. (**E**) The activity of lactic acid dehydrogenase (LDH) in the cells. n = 12 per group. The mice were injected (s.c.) with solvent (Ctrl) or DHEA for 20 consecutive days. (**F**) The lactic acid concentrations in the ovarian supernatants. n = 4 per group. (**G**) The ovarian LDH activity in the mice. n = 4 per group. Data are presented as mean ± SEM. *, *p* < 0.05, **, *p* < 0.01.

In our previous study, we observed that treating KGN cells with 50 μM DHEA for 24 h unexpectedly induced extracellular acidification. The pH of the culture medium from cells with or without DHEA treatment was thus quantified using a blood gas analyzer. As shown in Figure 1A, although the pH values remained within the neutral range, DHEA treatment significantly reduced extracellular pH (*p* = 0.0371), indicating the increased medium acidity. Intracellular pH was measured using BCECF AM, a widely used membrane-permeable fluorescent probe that is converted to BCECF within the cells and exhibits increased green fluorescence at higher pH. After DHEA treatment, KGN cells showed significantly lower fluorescence intensity than Ctrl cells (*p* = 0.001) (Figure 1B), indicating decreased cytoplasmic pH. Moreover, exposure of KGN cells to a series of DHEA concentrations revealed that 50 μM DHEA induced a significant decrease in cell viability compared with the control group (*p* < 0.0001), as detected by MTT (Supplementary Figure S1). Therefore, 50 μM DHEA was selected in subsequent experiments.

We next measured the levels of intracellular lactic acid, one of the key sources of H^+^. DHEA treatment significantly increased intracellular lactic acid concentrations (*p* = 0.0002) (Figure 1C), which subsequently led to elevated extracellular lactic acid levels compared with Ctrl group (*p* < 0.0001) (Figure 1D). Although DHEA treatment did not significantly upregulate lactic acid dehydrogenase (LDH) mRNA expression (*p* = 0.62) (Supplementary Figure S2A), it markedly enhanced LDH enzymatic activity (*p* < 0.0001) (Figure 1E), likely driving the observed lactic acid accumulation.

To investigate whether androgen excess induces lactic acid-related effects in ovarian GCs in vivo, we measured ovarian fluid lactic acid levels in a DHEA-induced PCOS mouse model. Our results demonstrated significantly elevated lactic acid concentrations in ovarian supernatants from PCOS mice (*p* = 0.0013) (Figure 1F). While LDH mRNA expression remained unchanged (*p* = 0.38) (Supplementary Figure S2B), the enzymatic activity of LDH in the ovaries was enhanced (*p* = 0.0166) (Figure 1G).

Collectively, these results demonstrated that hyperandrogenism disrupts pH homeostasis in both intracellular and extracellular compartments of ovarian GCs, with lactic acid playing a significant role in increasing relative acidification.

### 3.2. Intracellular Acidification Promotes Granulosa Cell Apoptosis and Contributes to Ovarian Impairment in PCOS

**Figure 2:**
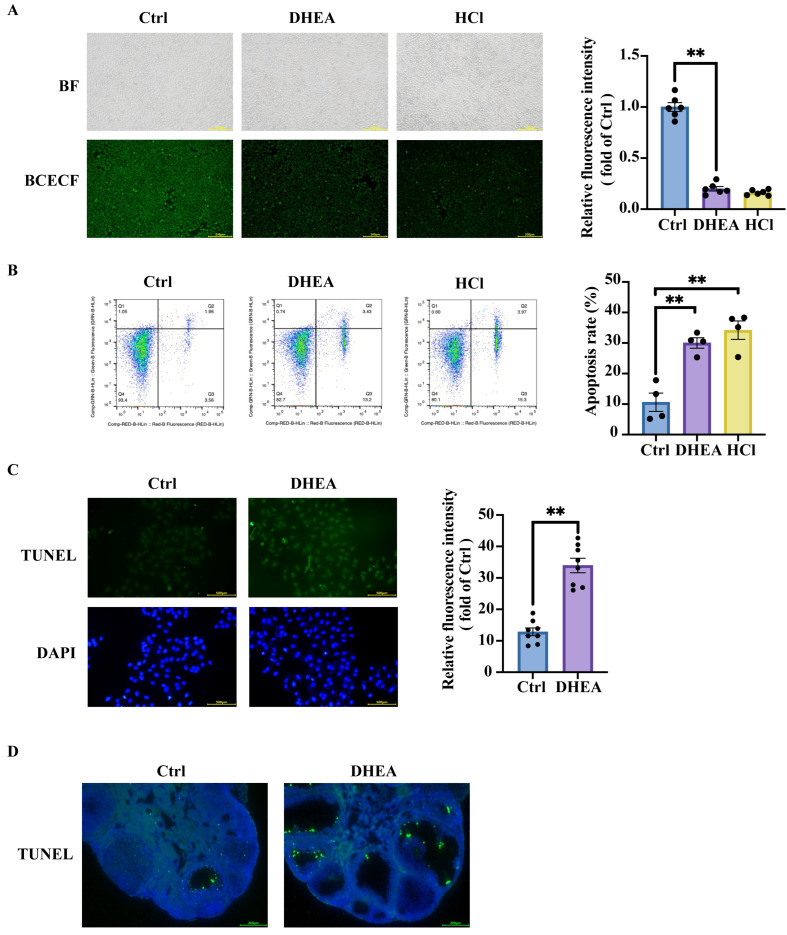
Intracellular acidification promotes granulosa cell apoptosis and ovarian impairment in PCOS. KGN cells were treated with the solvent (Ctrl) for 24 h, DHEA (50 μM) for 24 h, or HCl (10 mM) for 10 min. (**A**) Representative intracellular pH determined by BCECF and the statistical analysis of fluorescence intensity. Scale bar = 200 μm. n = 6 per group. BF: bright field. (**B**) Representative flow cytometry analysis to determine the percentage of cell apoptosis and the statistical analysis of apoptosis rate. n = 4 per group. (**C**) KGN cells were treated with the solvent (Ctrl) for 24 h or DHEA (50 μM) for 24 h. Representative TUNEL staining to measure the cell apoptosis and the statistical analysis of fluorescence intensity. Scale bar = 500 μm. n = 8 per group. (**D**) Representative TUNEL + DAPI staining of ovarian sections of one mouse from each group. TUNEL (green) determined apoptotic cells and DAPI (blue) marked the cell nucleus. Scale bar = 200 μm. Data are presented as mean ± SEM. **, *p* < 0.01.

The intracellular pH (pHi) is vital for most biological reactions, the structure and conformation of macromolecules, and protein interactions [22]. The chemical reactions of organisms are sensitive to pH, and the enzymes that facilitate these biological reactions have pH optima [23]. It has been demonstrated that extracellular pH (pHe) levels profoundly affect membrane transporters and lead to pHi fluctuation in the same direction [23]. To verify the impact of the acidification of the extracellular environment on cell survival, we treated KGN cells with a series of hydrochloric acid (HCl) at 0, 1, 10, and 100 mM, respectively. BCECF assays demonstrated that increasing HCl concentrations in the culture medium led to progressive intracellular acidification (Supplementary Figure S3A) [20]. The subsequent flow cytometry analysis revealed that decreased intracellular pH promoted cell apoptosis (Supplementary Figure S3B). Both DHEA treatment for 24 h and HCl for 10 min significantly reduced pHi in KGN cells (*p* < 0.0001) (Figure 2A), accompanied by increased apoptosis compared with controls (Ctrl vs. DHEA *p* = 0.0017; Ctrl vs. HCl *p* = 0.0004) (Figure 2B). These data suggested that cytoplasmic acidification contributes to cell apoptosis. The increased apoptosis of DHEA-treated KGN cells was further confirmed by TUNEL staining (*p* < 0.0001) (Figure 2C). Notably, PCOS mice exhibited increased apoptosis of ovarian GCs (Figure 2D), which may be partially attributed to the elevated ovarian lactic acid accumulation mentioned earlier. Together, these data demonstrate that decreased intracellular or extracellular pH promotes cellular apoptosis. Elevated ovarian lactic acid levels in PCOS mice contribute to the upregulated granulosa cell apoptosis in PCOS.

### 3.3. Hyperandrogenemia Contributes to Mitochondria Damage and Promotes Anaerobic Glycolysis, Leading to Lactic Acid Accumulation in KGN Cells

**Figure 3:**
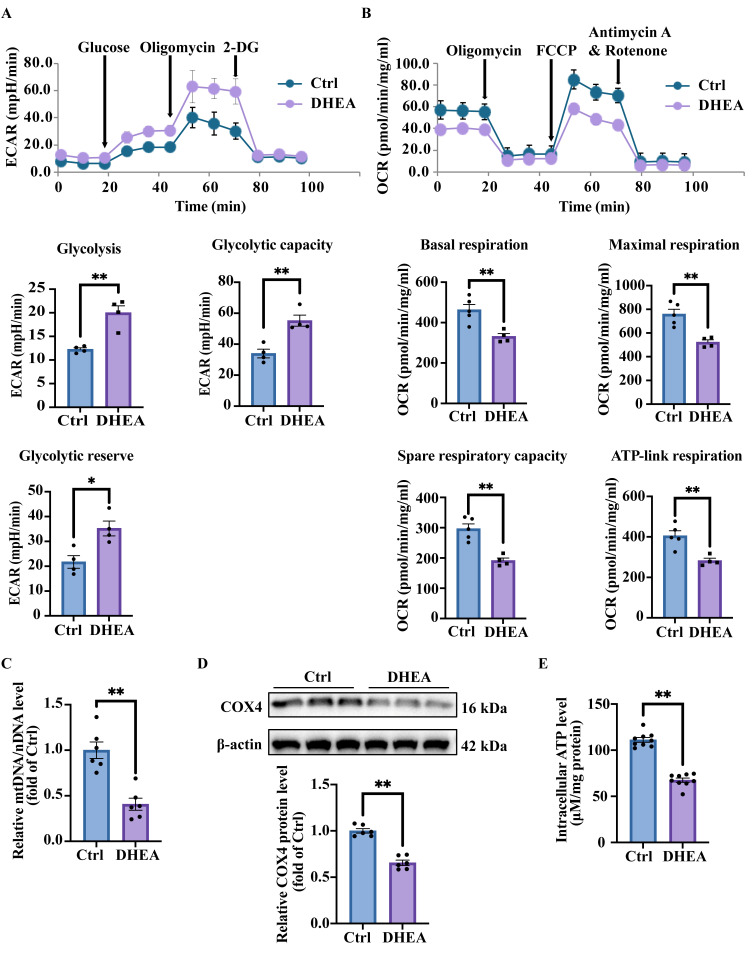
DHEA induces mitochondrial damage, anaerobic glycolysis, and lactic acid accumulation in KGN cells. KGN cells were treated with DHEA (0, 50 μM) for 24 h. (**A**) Representative experiment to determine extracellular acidification rate (ECAR) of cells in response to different glycolytic substrates or inhibitors. ECAR was measured by a Seahorse XF24 analyzer, with adding reagents at the indicated time points. Statistical analysis of the glycolysis, glycolytic capacity and glycolytic reserve were also presented. n = 4 per group. (**B**) Representative experiment to determine oxygen consumption rate (OCR) of cells in response to different mitochondrial respiration inhibitors. OCR was measured by a Seahorse XF24 analyzer, with adding reagents at the indicated time points. Quantification of the basal respiration, maximal respiration, spare respiration and ATP-linked OCR were analyzed were also presented. n = 4 per group. (**C**) The relative ratio of mitochondrial DNA (ND1) to nuclear DNA (SLCO2B1) mRNA levels to represent mitochondrial DNA copy number. n = 6 per group. (**D**) Western blot analysis and densitometry quantification of COX4 protein expression to represent the amount of mitochondria. n = 6 per group. (**E**) Normalized intracellular ATP production of the cells. n = 6 per group. Data are presented as mean ± SEM. *, *p* < 0.05, **, *p* < 0.01.

Intracellular lactic acid originates from glycolysis, glutamine decomposition, cell-to-cell shuttling (via non-channel pathways or monocarboxylate transporters), etc. [24]. To determine the cause of lactic acid accumulation, we assessed the respiratory capacity of cells using the Seahorse XF Extracellular Flux Analyzer. Extracellular acidification rate (ECAR) is widely used to evaluate glycolytic activity in live cells. We thus measured ECAR under various treatment conditions. KGN cells were treated with 50 μM DHEA for 24 h. Cells treated with the solvent were defined as Ctrl. Then the cells were sequentially treated with glucose, the ATP synthase inhibitor oligomycin, and the glycolytic pathway inhibitor 2-DG. Glycolytic function was then assessed by measuring ECAR, including basal glycolysis, glycolytic capacity, and glycolytic reserve. As shown in Figure 3A, DHEA treatment significantly enhanced glycolysis in KGN cells (*p* = 0.0023). The pronounced upregulation of anaerobic respiration likely accounts for the observed accumulation of intracellular lactic acid. Furthermore, DHEA treatment also significantly enhanced both the glycolytic capacity (*p* = 0.0033) and glycolytic reserve (*p* = 0.0137) of cells.

A representative oxygen consumption rate (OCR) curve of the cells treated in the presence or absence of DHEA was shown in Figure 3B. The basal respiration showed energetic demand of cells under baseline conditions. Oligomycin significantly reduced OCR when administered. Subsequent treatment with the mitochondrial uncoupler FCCP disrupted the proton gradient across the inner mitochondrial membrane, resulting in maximal OCR. The spare respiratory capacity, defined as the difference between maximal and basal respiration, reflects the cellular ability to meet increased energy demands. Treatment with the electron transport chain inhibitors rotenone and antimycin A completely suppressed mitochondrial respiration, reducing OCR to minimal levels. As illustrated in Figure 3B, the DHEA-treated group exhibited significantly lower OCR values for basal respiration (*p* = 0.0059), ATP-linked respiration (*p* = 0.0051), maximal respiration (*p* = 0.0024), and spare respiratory capacity (*p* = 0.0012) compared with Ctrl group. These data demonstrated that DHEA treatment impaired aerobic respiration in KGN cells and reduced their ability to respond to ATP demands. These data suggested that the observed increase in anaerobic respiration may represent a compensatory mechanism for inadequate energy production through oxidative phosphorylation. DHEA treatment increased anaerobic respiration.

Mitochondria serve as the exclusive sites of aerobic respiration in eukaryotic cells, while anaerobic respiration (glycolysis) takes place in the cytosol independently of mitochondria. The shift in respiratory preference in DHEA-treated KGN cells may be attributed to the reduction in mitochondria, which is essential for oxidative phosphorylation. To quantify the mitochondrial content, we measured relative levels of mitochondrial-specific markers in KGN cells treated with or without DHEA, including mtDNA ND1 and nDNA SLCO2B1. The results demonstrated that DHEA treatment significantly reduced the mtDNA copy number, as quantified by the mtDNA/nDNA ratio (*p* = 0.0004) (Figure 3C). The protein expression of another mitochondrial protein COX4 was also examined. Consistent with the mtDNA copy number, we observed a marked downregulation of COX4, a mitochondrial marker protein, in DHEA-treated cells (*p* < 0.0001) (Figure 3D). Intracellular ATP quantification also revealed a significant reduction in DHEA-treated cells (*p* < 0.0001) (Figure 3E), further confirming the mitochondrial dysfunction in DHEA-treated cells.

Taken together, these findings demonstrate mitochondrial dysfunction and a shift in cellular energy metabolism in KGN cells treated with DHEA. Our data demonstrate that mitochondrial dysfunction drives a metabolic shift toward anaerobic respiration, leading to lactic acid accumulation and intracellular acidification.

### 3.4. Mitochondria Damage Also Triggers Granulosa Cell Apoptosis Through Acidification—Independent Pathways

In addition to modulating cell apoptosis through cytoplasmic acidification, mitochondrial damage may also activate apoptosis-inducing factor (AIF)-dependent apoptotic signaling pathways. AIF, normally localized within the mitochondrial intermembrane space, is released into the cytosol upon mitochondrial damage and subsequently translocases to the nucleus via nuclear pores. Western blot analysis revealed a significant increase in AIF protein expression in the mitochondrial-free cytoplasmic fraction of DHEA-treated cells compared with Ctrl cells (Supplementary Figure S4A). VDAC1 was used as a mitochondrial protein marker in this experiment. Consistent with these findings, immunofluorescence assays further demonstrated the translocation of AIF in DHEA-treated cells (*p* < 0.0001) (Supplementary Figure S4B). Additionally, nuclear proteins were extracted and Lamin B1 was used as a nuclear protein marker. Western blot analysis further confirmed increased AIF expression in the nuclear fraction of DHEA-treated cells (*p* = 0.0003) (Supplementary Figure S4C). Results from the comet assay also demonstrated DNA damage in DHEA-treated cells, suggesting an increase in cell death (Supplementary Figure S4D). Collectively, these data demonstrate that DHEA treatment induces AIF release from mitochondria, leading to DNA fragmentation and ultimately triggering cell apoptosis.

To determine whether these effects were mediated by androgen receptor (AR) activation, we co-treated KGN cells with DHEA and the AR antagonist Flutamide (Flu) across a concentration gradient. MTT assays showed that flutamide ameliorated DHEA-induced cell apoptosis in a dose-dependent manner, indicating a pivotal role for AR in this process (Supplementary Figure S5).

### 3.5. Oxidative Stress in DHEA-Treated KGN Cells and Mouse Ovaries

**Figure 4:**
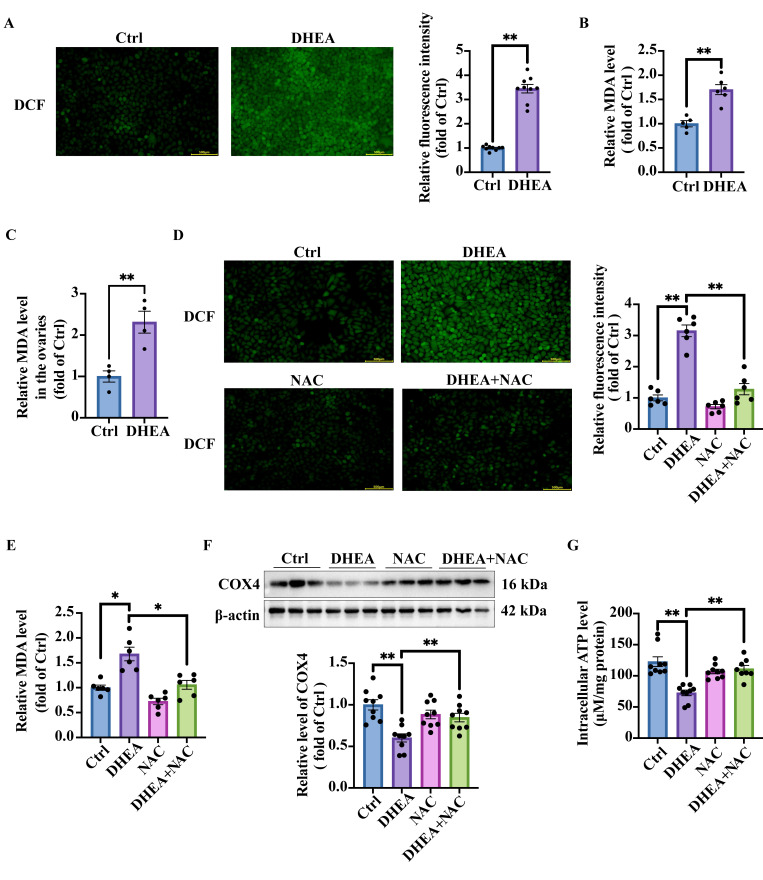
NAC alleviates DHEA-induced oxidative stress and mitochondrial dysfunction in KGN cells. KGN cells were treated with DHEA (0, 50 μM) for 24 h. (**A**) Representative fluorescence micrographs of intracellular DCF indicating ROS in the cells. Scale bar = 500 μm. The relative intensity of fluorescence was analyzed. n = 9 per group. (**B**) The relative intracellular MDA concentrations. n = 6 per group. (**C**) The mice were injected (s.c.) with the solvent (Ctrl) or DHEA for 20 consecutive days. The relative ovarian MDA concentrations in the mice. n = 4 per group. (**D**) KGN cells were pre-treated with NAC (0, 10 mM) for 1 h, followed by the treatment with the solvent (Ctrl), 50 μM DHEA (DHEA), 10 mM NAC (NAC), or DHEA + NAC for 24 h. Representative fluorescence micrographs of intracellular DCF indicating ROS in cells. Scale bar = 500 μm. The relative intensity of fluorescence was analyzed. n = 6 per group. (**E**) The relative intracellular MDA concentrations. n = 6 per group. (**F**) Western blot analysis and densitometry quantification of COX4 protein expression to represent the amount of mitochondria. n = 9 per group. (**G**) Normalized intracellular ATP production of the cells. n = 9 per group. Data are presented as mean ± SEM. *, *p* < 0.05, **, *p* < 0.01.

Mitochondria are the primary source of oxidative stress and mitochondrial damage can exacerbate oxidative stress. Conversely, oxidative stress can also impair mitochondrial function [25]. DCFH-DA fluorescent probe was thus used in KGN cells treated with or without DHEA to measure reactive oxygen species (ROS). Intracellular ROS oxidized the non-fluorescent DCFH into fluorescent DCF, enabling the quantification of ROS levels based on fluorescence intensity. As shown in Figure 4A, DHEA-treated group exhibited significantly higher fluorescence intensity than the Ctrl group (*p* < 0.0001), suggesting cellular oxidative stress. We further measured other oxidative stress markers, including the intracellular malondialdehyde (MDA) content and superoxide dismutase (SOD) activity. MDA levels were significantly increased in DHEA-treated cells (*p* = 0.0004) (Figure 4B), further confirming oxidative stress in DHEA-treated cells. The SOD activity, however, was also increased in DHEA group (*p* = 0.0004) (Supplementary Figure S6A). We have previously documented comparable SOD activity elevation in skeletal muscle of PCOS mice, indicating that this may be an adaptive antioxidant compensation effect [15]. We next assessed whether PCOS mouse ovaries exhibited oxidative stress. Consistent with the in vitro findings, ovarian MDA levels were significantly elevated in DHEA-induced PCOS mice (*p* = 0.0046) (Figure 4C), confirming oxidative stress. The concomitant rise in SOD activity suggests activation of compensatory antioxidant defenses (*p* < 0.0001) (Supplementary Figure S6B). In summary, our data demonstrate that androgen excess induces oxidative stress in GCs, establishing a feed-forward loop wherein oxidative stress and mitochondrial dysfunction mutually exacerbate each other.

### 3.6. NAC Targets Oxidative Stress and Ameliorates Androgen Excess-Induced Mitochondrial Damage in KGN Cells

To further validate the reciprocal relationship between oxidative stress and mitochondrial damage, an antioxidant agent NAC was used in cell experiments. DCF fluorescence imaging demonstrated a substantial increase in intracellular ROS levels in DHEA-treated cells. However, the co-treatments of cells with DHEA and NAC significantly reduced ROS accumulation compared with DHEA treatment alone (*p* < 0.0001). Notably, NAC treatment alone did not have any detectable effect on basal ROS production (*p* > 0.9999) (Figure 4D). Intracellular MDA levels exhibited a trend similar to ROS (Ctrl vs. DHEA *p* = 0.0006; DHEA vs. DHEA + NAC *p* = 0.0015) (Figure 4E). NAC showed antioxidant efficacy by mitigating DHEA-induced mitochondrial damage, as evidenced by significantly higher expression of the mitochondrial marker COX4 in NAC-treated cells compared with DHEA treatment alone (Ctrl vs. DHEA *p* < 0.0001; DHEA vs. DHEA + NAC *p* = 0.011) (Figure 4F), indicating the recovery of mitochondrial quantity. Consistent with our earlier findings, DHEA-treated cells exhibited significantly reduced ATP production (*p* < 0.0001). In contrast, DHEA + NAC treatment restored ATP generation to levels comparable to the Ctrl group (*p* = 0.0006) (Figure 4G). Notably, NAC alone showed no effect on mitochondrial parameters, as both COX4 expression and ATP production remained unchanged compared with the Ctrl group (*p* > 0.9999) (Figure 4F,G). In summary, NAC treatment protected GCs from androgen excess-induced mitochondrial damage.

### 3.7. NAC Reduces Lactic Acid Accumulation, Ameliorates Intracellular pH, and Decreases Cell Apoptosis

**Figure 5:**
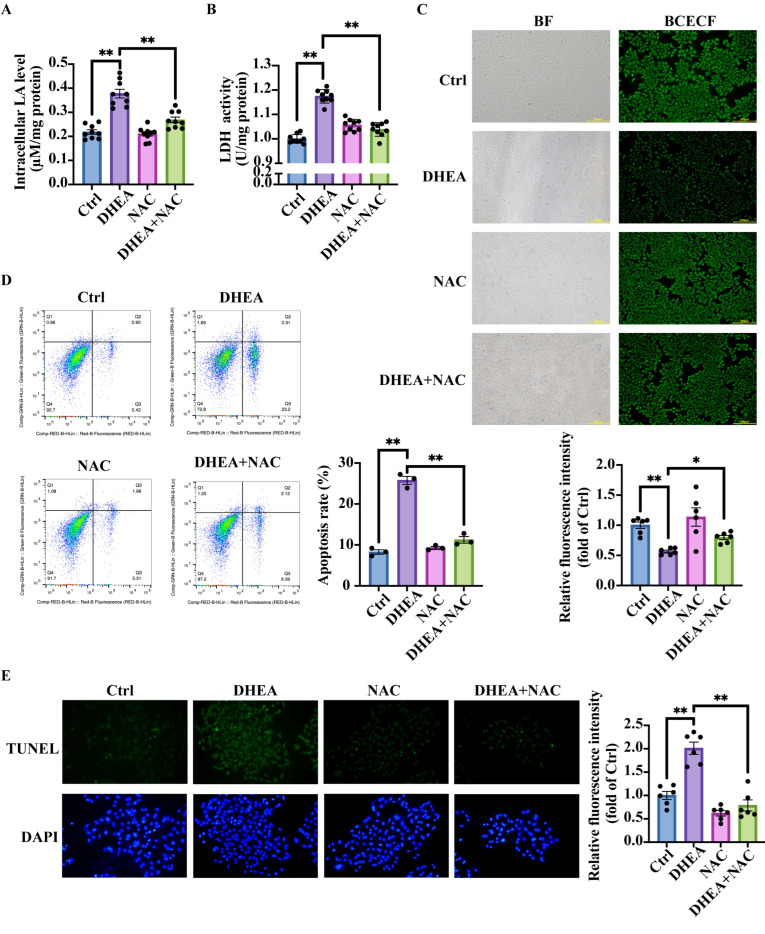
NAC reduces lactic acid levels and improves DHEA-induced intracellular acidification, thus decreasing apoptosis in KGN cells. KGN cells were pre-treated with NAC (0, 10 mM) for 1 h, followed by the treatment with the solvent (Ctrl), 50 μM DHEA (DHEA), 10 mM NAC (NAC) or DHEA + NAC for 24 h. (**A**) The intracellular lactic acid (LA) concentrations. n = 9 per group. (**B**) The intracellular lactic acid dehydrogenase (LDH) activity. n = 9 per group. (**C**) Representative fluorescence micrographs of the intracellular pH determined by BCECF and the statistical analysis of fluorescence intensity. n = 6 per group. (**D**) Representative flow cytometry analysis to determine the percentage of cell apoptosis and the statistical analysis of apoptosis rate. n = 3 per group. (**E**) Representative TUNEL staining to measure the cell apoptosis and the statistical analysis of fluorescence intensity. n = 6 per group. Data are presented as mean ± SEM. *, *p* < 0.05, **, *p* < 0.01.

We next investigated whether NAC could suppress the mitochondrial damage-induced lactic acid accumulation. Compared with Ctrl group, DHEA-treated cells showed significantly elevated intracellular lactic acid levels (*p* < 0.0001), an effect attenuated by NAC co-treatment to Ctrl-equivalent levels (*p* < 0.0001) (Figure 5A). The corresponding decreases in LDH activity were observed in the DHEA + NAC group compared with the DHEA group (*p* < 0.0001) (Figure 5B). The decreased lactic acid content seemed to mediate intracellular pH recovery. BCECF fluorescence imaging revealed that DHEA + NAC treatment restored intracellular pH to Ctrl levels, as evidenced by ameliorated fluorescence intensity in DHEA + NAC-treated cells compared with DHEA-treated cells (*p* < 0.0001) (Figure 5C). There was no obvious difference in the fluorescence intensity between the DHEA + NAC group and the Ctrl group (*p* = 0.0693).

Since NAC improves mitochondrial function and reduces lactic acid accumulation, we next examined whether NAC could attenuate cell apoptosis. The TUNEL assay was thus performed to detect the apoptosis of KGN cells in four groups: Ctrl, DHEA, NAC, and DHEA + NAC. Results from flow cytometry revealed that DHEA significantly increased the number of apoptotic cells, whereas treatment of cells with DHEA + NAC markedly reduced cell apoptosis (Ctrl vs. DHEA *p* < 0.0001; DHEA vs. DHEA + NAC *p* < 0.0001) (Figure 5D). The TUNEL assay results were consistent with flow cytometry analysis, as illustrated in Figure 5E (Ctrl vs. DHEA *p* < 0.0001; DHEA vs. DHEA + NAC *p* < 0.0001).

In summary, exposure of KGN cells to high androgen levels induced oxidative stress and mitochondrial dysfunction. Antioxidant therapy effectively reduced oxidative stress and mitochondrial damage, alleviated intracellular acidosis, and ultimately decreased cell apoptosis.

### 3.8. NAC Mitigates Lactic Acid Accumulation and Restores Ovarian Morphology in PCOS Mice

**Figure 6:**
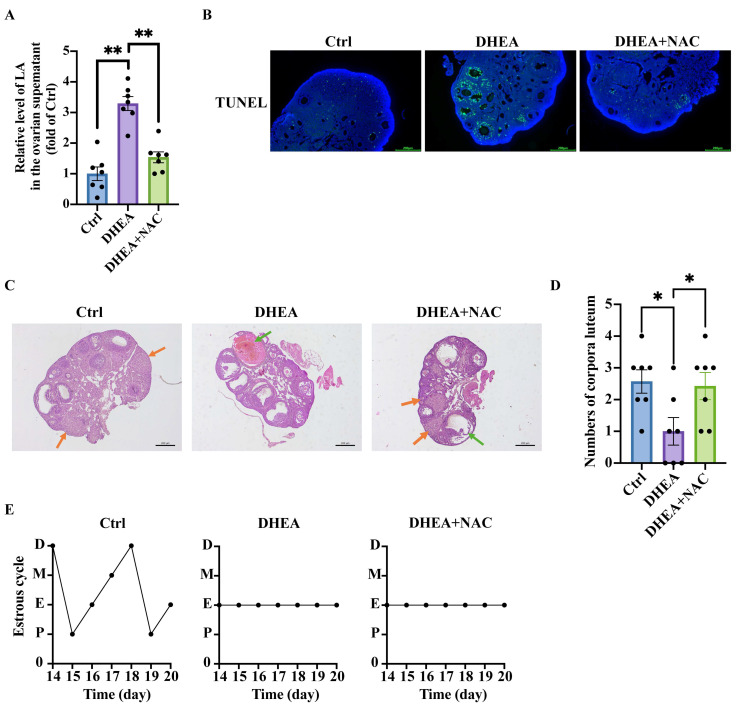
Antioxidant NAC mitigates lactic acid accumulation and restores ovarian morphology in PCOS mice. The mice were treated with the solvent (Ctrl) (s.c.), DHEA (s.c.)+ saline (i.g.) (DHEA), or DHEA (s.c.) + NAC solution (i.g.) (DHEA + NAC) for 20 consecutive days. (**A**) The relative lactic acid (LA) concentrations in the ovarian supernatants. n = 7 per group. (**B**) Representative TUNEL + DAPI staining of ovarian sections of one mouse from each group to evaluate the cell apoptosis. TUNEL (green) determined apoptotic cells and DAPI (blue) marked the cell nucleus. Scale bar = 200 μm. (**C**) Representative H&E staining of ovarian sections of one mouse from each group. Scale bar = 200 μm. (**D**) The number of corpora luteum of ovaries harvested from the mice in different group. n = 7 per group. (**E**) Representative estrous cycle of one mouse from each group. D, diestrus; M, metestrus; E, estrus; P, proestrus. Data are presented as mean ± SEM. *, *p* < 0.05, **, *p* < 0.01.

Based on the in vitro findings, we administered NAC to DHEA-induced PCOS mice via intragastric gavage to evaluate its effects on ovarian function. As expected, the lactic acid levels in the ovarian supernatant of DHEA + NAC mice were significantly lower than those in DHEA animals (Ctrl vs. DHEA *p* < 0.0001; DHEA vs. DHEA + NAC *p* < 0.0001) (Figure 6A). TUNEL staining of mouse ovarian sections showed the increased apoptotic GCs in the DHEA group compared with Ctrl group (Figure 6B). As expected, the apoptotic GCs in the DHEA + NAC mice were lower than DHEA mice, as indicated by the reduced green fluorescence (Figure 6B). Histological analysis of mouse ovaries revealed that DHEA-treated mice showed typical polycystic ovarian morphology, marked by the presence of multiple cystic follicles. In contrast, DHEA + NAC mice showed normal ovarian morphology, exhibiting no significant cystic formation (Figure 6C). The corpus luteum is a transient endocrine structure derived from post-ovulatory follicles, with the number of corpora lutea serving as a key indicator of ovarian ovulatory ability. The DHEA-induced PCOS mice exhibited significantly fewer corpora lutea than Ctrl, while NAC supplementation partially restored luteal formation in DHEA-treated mice (Ctrl vs. DHEA *p* = 0.0177; DHEA vs. DHEA + NAC *p* = 0.0376) (Figure 6D). These data suggested that NAC partially improved ovarian function in PCOS mice.

Meanwhile, assessment of the estrous cycle showed that Ctrl mice maintained normal cyclicity, whereas DHEA-induced PCOS mice exhibited persistent estrus arrest. Notably, NAC co-treatment failed to restore regular estrous cyclicity in DHEA-treated mice (Figure 6E).

## 4. Discussion

The present study demonstrated that DHEA-induced PCOS mice exhibited elevated serum and ovarian lactic acid levels. High androgen exposure induces lactic acid accumulation driven by mitochondrial dysfunction and enhanced anaerobic glycolysis, leading to acidification of both the extracellular and intracellular environments and ultimately cell apoptosis in both ovarian granulosa cells of PCOS mice and in vitro cultured KGN cells. NAC, an antioxidant that reduces oxidative stress and ameliorates mitochondrial damage in GCs, decreased lactic acid production, restored the intracellular pH value, and mitigated cell apoptosis. These findings indicate that hyperandrogenism-induced lactic acid accumulation represents one of the mechanisms underlying granulosa cell apoptosis in PCOS. Additionally, our data provide a rationale for exploring NAC as a potential auxiliary treatment for PCOS-associated ovarian dysfunction in clinical practice.

PCOS is a common gynecological endocrine and metabolic disorder that not only affects reproductive function but is also closely related to a variety of metabolic abnormalities. Many studies have examined alterations in glucose metabolism in PCOS patients. Elevated circulating lactic acid levels were reported in women with PCOS by non-targeted metabolomics studies [17,18]. RNA sequencing (RNA-seq) analysis of GCs isolated from ovaries of PCOS patients revealed significant enrichment of differentially expressed genes in the glycolysis/gluconeogenesis pathways [26]. Moreover, primary GCs from PCOS patients exhibited enhanced lactic acid production and a significantly higher glycolysis index compared with the Ctrl group [27].

Conflicting evidence exists regarding lactic acid levels in PCOS. Some studies reported no significant differences or even slightly decreased lactic acid concentrations in PCOS patients compared with controls [28,29]. This discrepancy may be attributed to whether the patients were obese. A nuclear magnetic resonance (NMR)-based metabolomic analysis [18] revealed that the most pronounced metabolic alterations in PCOS women include elevated lactic acid levels. The significantly changed metabolic markers clearly distinguished non-obese PCOS patients from healthy controls. However, this discriminatory pattern was markedly attenuated in the obese subgroups, where differences in lactic acid were no longer significant. Currently, it remains unknown whether ovarian lactate levels are altered in PCOS patients or what impact such changes might have on ovarian function.

In animal studies, Liu et al. [28,29] found that ovarian lactic acid content was reduced by approximately 50% in letrozole-induced, high-fat diet (HFD)-fed PCOS rats compared with controls. In contrast, our current study demonstrated a significant increase in ovarian lactic acid levels in DHEA-induced PCOS mice. The discrepancy may be due to the pronounced obesity in letrozole + HFD-induced experimental animals. However, both Liu et al.’s findings and our results demonstrate that altered lactate levels are associated with GC apoptosis.

The mechanism underlying elevated ovarian lactic acid levels in PCOS remains controversial. Substantial evidence suggests this phenomenon is unlikely to be mediated by the direct effects of androgens. It has been reported that low-dose androgens significantly potentiated the response to follicle-stimulating hormone (FSH), resulting in a 2-fold increase in lactic acid levels following 48-h co-treatment with FSH compared with untreated controls [30]. Some studies reported that insulin activates the PI3K/Akt signaling pathway in ovarian GCs and stimulates lactic acid production, whereas hyperandrogenism appears to exert minimal influence on both insulin signaling and metabolic effects in GCs [31]. In vitro studies using primary rat ovarian GCs demonstrated that testosterone and 5α-dihydrotestosterone (10^−8^ to 10^−6^ M) had no direct effect on basal lactic acid accumulation, which is different from our findings. This discrepancy is probably due to the difference in androgen concentrations, as we used 50 μM DHEA in the current study.

Several studies have identified elevated plasma lactic acid as a biomarker of disease severity and cellular stress response [32]. Energy stress is a complex process that causes and participates in the reprogramming of various metabolic processes. Anaerobic respiration often occurs in skeletal muscle and brain cells. These cells are thus most susceptible to intracellular lactic acid accumulation under stress, leading to cell apoptosis and organ dysfunction [33,34]. In the present study, DHEA can promote glycolysis in KGN cells. The abnormal accumulation of lactic acid through glycolysis is also closely related to cell apoptosis of GCs due to its properties as an organic acid. The lactic acid-mediated acidic environment promotes MDH1-catalyzed reduction of α-ketoglutarate to L-2HG, thereby modulating caspase-8-GSDMC-dependent cell pyroptosis [35]. At pH = 6.5, TNF-α induces a caspase-8-dependent apoptosis rather than necrosis. The acidic environment promotes the cleavage and nuclear translocation of AIF, mediating cell apoptosis [36]. These findings have been consistently validated in ovarian GCs in the current work.

Additionally, mitochondrial dynamics disruption has been identified as a potential mechanism underlying lactic acid-mediated cell death. Excessive glycolytic metabolism of neutrophils contributes to the formation of neutrophil extracellular traps (NEPs), which is a beneficial suicidal death by neutrophils. Exogenous lactic acid treatment can also induce NETosis in neutrophils, playing an unexpected role in experimental models of sepsis [37]. Meanwhile, growing evidence suggests that lactic acid plays a significant role in protein post-translational modification (PTM). It has been demonstrated that aberrant protein lactylation can trigger detrimental cellular consequences. For instance, lactic acid from the hepatocyte microenvironment mediates NEDD4 lactylation, which reduces Caspase-11 ubiquitination by weakening the NEDD4–Caspase-11 interaction. This pathway modulates atypical apoptosis of macrophages and promotes acetaminophen-induced acute liver injury [38]. Moreover, the pro-apoptotic effects of lactic acid extend to other pathophysiological contexts through distinct pathways. In pulmonary fibrosis, lactic acid exacerbates disease progression by activating the ATF4/CHOP axis and Caspase-12, which induces endoplasmic reticulum stress and apoptosis in alveolar epithelial cells [39].

In PCOS, GCs exhibit mitochondrial dysfunction and elevated oxidative stress, which promote GC apoptosis and ultimately contribute to impaired folliculogenesis [40]. Excessive ROS induces oxidative damage to cellular components, further accelerating apoptosis and follicular atresia [41]. Our findings support the mechanism that hyperandrogenemia-induced mitochondrial damage increased glycolytic flux and lactic acid accumulation, leading to intracellular acidification and GC apoptosis. Consistent with our previous results showing that alleviating cellular stress reduces GC apoptosis in PCOS [14], the current study underscores mitochondria as a key mediator in PCOS pathology. Future studies could focus on targeted strategies to restore mitochondrial integrity and redox homeostasis, which may offer a novel approach for improving ovarian function and fertility in PCOS [42].

Given that PCOS is a complex disorder with both endocrine and metabolic manifestations, it must be acknowledged that this study focused exclusively on hyperandrogenism as the driver of lactic acid accumulation and the subsequent granulosa cell apoptosis in PCOS. Therefore, future research should investigate the impact of metabolic syndrome-associated factors, such as obesity, hyperinsulinemia, and insulin resistance, on this pathogenic pathway.

In summary, this study revealed for the first time that the lactic acid accumulation-mediated acid-base disruption drives apoptosis in ovarian GCs under hyperandrogenic conditions. Hyperandrogenism can directly induce mitochondrial damage and changes in cellular respiratory patterns, leading to lactic acid accumulation and ultimately causing cell apoptosis of GCs. The antioxidant NAC can ameliorate mitochondrial function, attenuate circulating and ovarian lactic acid levels, and thus partially improve the ovarian function in DHEA-induced PCOS mice. These findings reveal previously unrecognized pathological mechanisms in PCOS and identify potential therapeutic targets for clinical intervention.

## Data Availability

The original contributions presented in this study are included in the article/Supplementary Material. Further inquiries can be directed to the corresponding author(s).

## Supplementary Materials

The following supporting information can be downloaded at: https://www.mdpi.com/article/10.3390/antiox14101235/s1, Figure S1: DHEA induces KGN cells apoptosis in a dose-dependent manner; Figure S2: DHEA treatment does not significantly affect LDH mRNA expression in granulosa cells; Figure S3: Treatment of KGN cells with HCl leads to the reduced intracellular pH and cell apoptosis; Figure S4: DHEA induces AIF translocation in KGN cells and mediates apoptosis independent of caspase signaling; Figure S5: AR antagonist improves DHEA-induced KGN cells apoptosis via a dose-dependent manner; Figure S6 DHEA increases SOD activity in both KGN cells and PCOS mouse ovaries; Uncropped blot images.
