# Supplementary material for: Androgen-Induced Lactic Acid Accumulation Contributes to the Apoptosis of Ovarian Granulosa Cells in Polycystic Ovary Syndrome Mice
Source: Antioxidants (Basel). 2025 Oct 14;14(10):1235. doi: 10.3390/antiox14101235 (PMC12561039; doi:10.3390/antiox14101235)
Supplement: Supplementary file 1 [file antioxidants-14-01235-s001.zip › antioxidants-3880570-supplementary.pdf]

Supplementary Materials

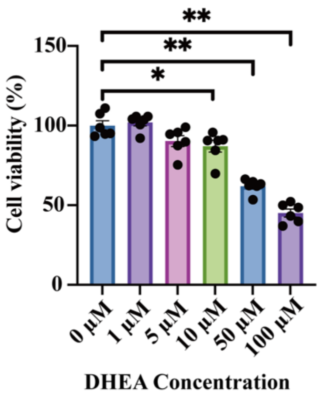

Supplementary Figure S1 DHEA induces KGN cells apoptosis in a dose-dependent manner. KGN cells were treated with DHEA (0, 1, 5, 10, 50 and 100 μM) for 24 hours. (A) Cell viability measured by MTT. n = 6 per group. Data are presented as mean ± SEM. *,p<0.05; **, p<0.01.

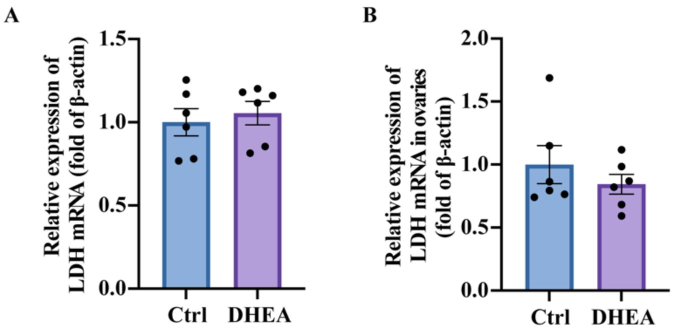

Supplementary Figure S2 DHEA treatment does not significantly affect LDH mRNA expression in granulosa cells. (A) KGN cells were treated with DHEA (0, 50 μM) for 24 hours. The relative mRNA expression of LDH in the cells detected by qRT-PCR. n = 6 per group. (B) The mice were injected (s.c.) with solvent (Ctrl) or DHEA for 20 consecutive days. The unilateral ovary of each mouse was used to extract total RNA. The relative mRNA expression of LDH in the ovaries detected by qRT-PCR. n = 6 per group. Data are presented as mean ± SEM.

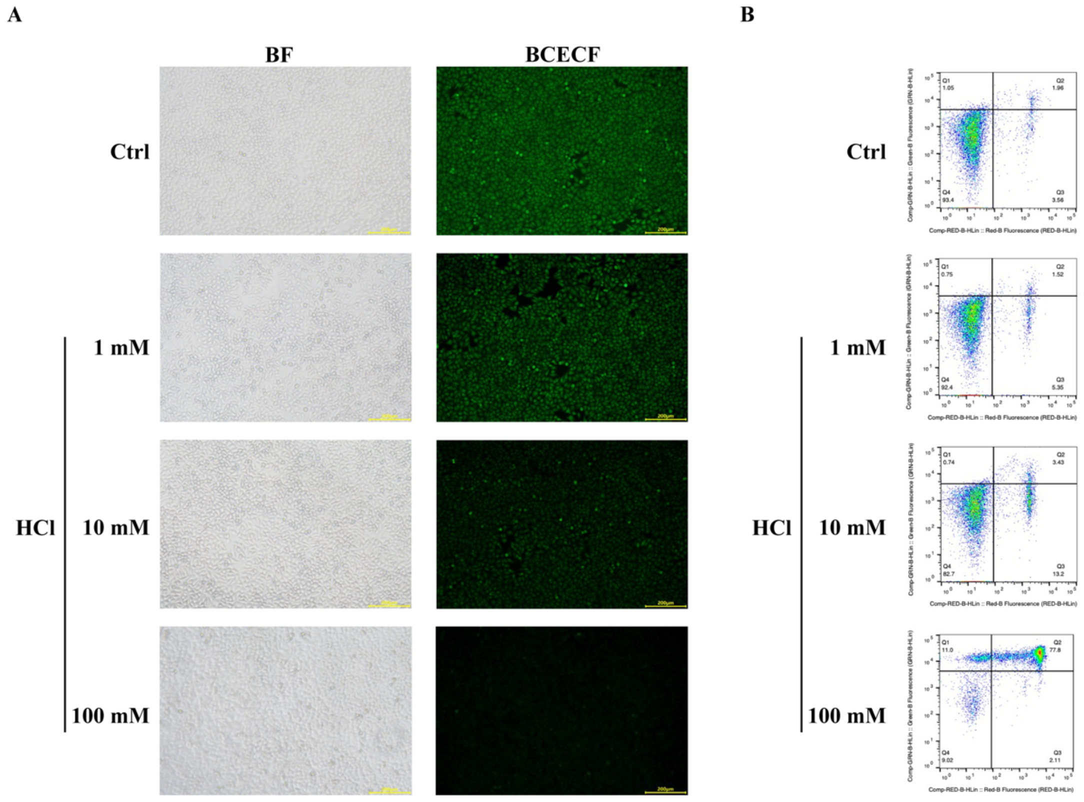

Supplementary Figure S3 Treatment of KGN cells with HCl leads to the reduced intracellular pH and cell apoptosis. KGN cells were treated with the solvent or different concentrations of HCl (1, 10, 100 mM) for 10 min. (A) Representative fluorescence micrographs of the intracellular pH determined by BCECF. BF: bright field. (B) The percentage of cell apoptosis measured by flow cytometry.

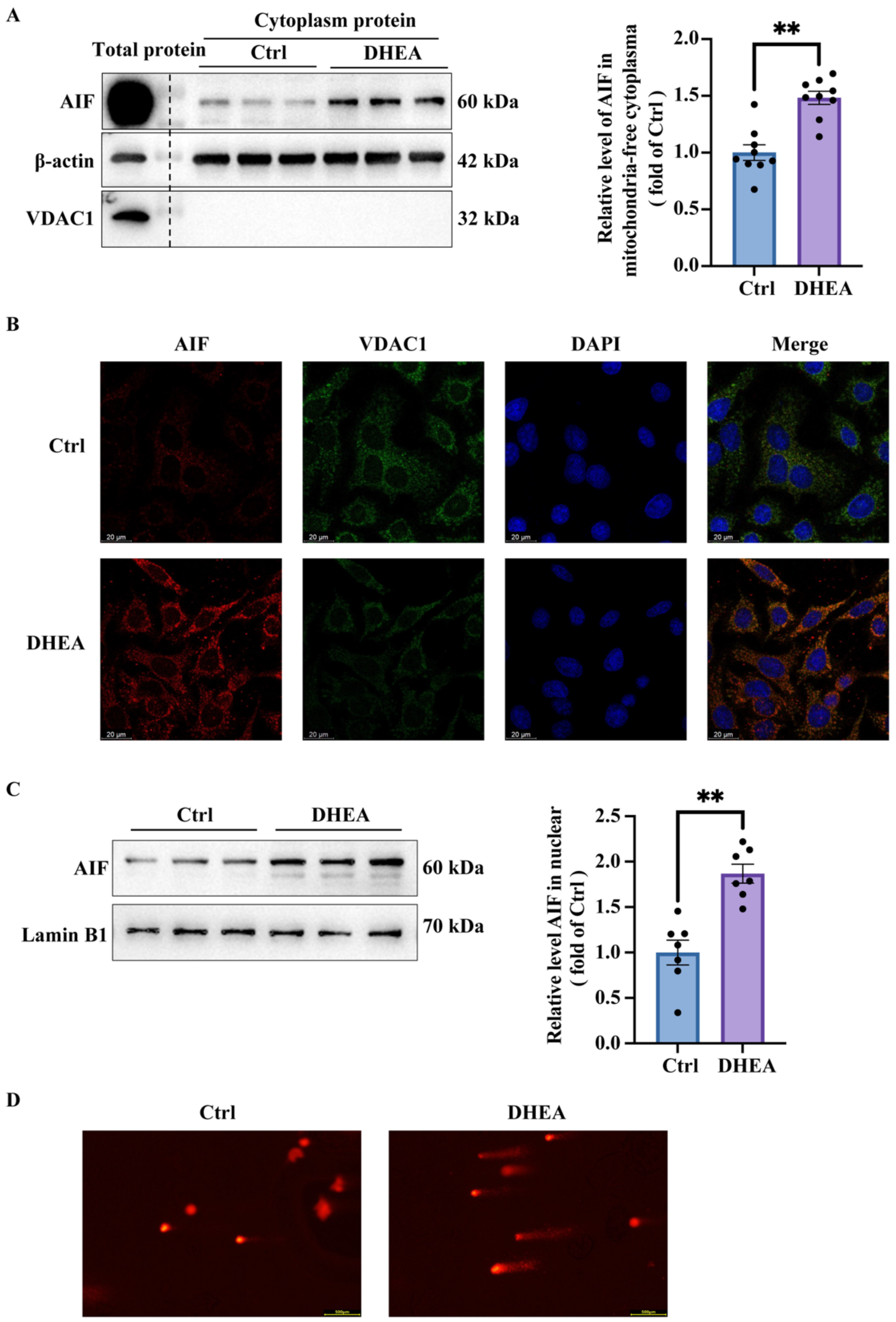

Supplementary Figure S4 DHEA induces AIF translocation in KGN cells and mediates apoptosis independent of caspase signaling. KGN cells were treated with DHEA (0, 50 μM) for 24 hours. (A) Western blot analysis and densitometry quantification of AIF in the mitochondria-free cytoplasm of KGN cells. β-actin was used as an internal control of cytoplasm protein. VDAC1 was used as a marker of mitochondria. n = 8 per group. (B) Representative immunofluorescence staining of KGN cells

permeabilized by digitonin. The cell membrane was punctured but the mitochondrial membrane was intact. So the antibody could not label proteins located inside the mitochondria. AIF is labeled in red and mitochondria (marked by VDAC1) are labeled in green. DAPI (blue) marked the cell nucleus. (C) Western blot analysis and densitometry quantification of AIF in the nucleus of KGN cells. Lamin B1 was used as an internal control of nuclear protein. VDAC1 was used as a marker of mitochondria. n = 7 per group. (D)The representative fluorescence picture of Comet assay showing DNA fragmentation. SYBR GOLD stained DNA fragments red and formed long tails. Data are presented as mean ± SEM. **, p<0.01.

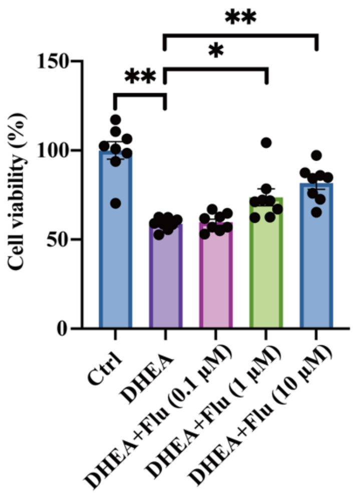

Supplementary Figure S5. AR antagonist improves DHEA-induced KGN cells apoptosis via a dose-dependent manner. KGN cells were treated with DHEA (0, 50 μM) and Flutamide (Flu) (0.1, 1, 10 μM) for 24 hours. (A) Cell viability measured by MTT. n = 8 per group.Data are presented as mean ± SEM. *, p<0.05; **, p<0.01.

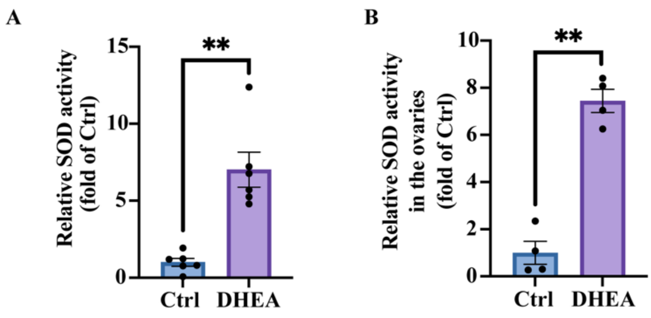

Supplementary Figure S6 DHEA increases SOD activity in both KGN cells and PCOS mouse ovaries. (A) KGN cells were treated with DHEA (0, 50 μM) for 24 hours. The relative activity of SOD in the cells. n = 6 per group. (B) The mice were injected (s.c.) with the solvent (Ctrl) or DHEA for 20 consecutive days. The relative activity of SOD in the ovaries of the mice. n = 4 per group. Data are presented as mean ± SEM. **, *p*<0.01.

Fig. 3D

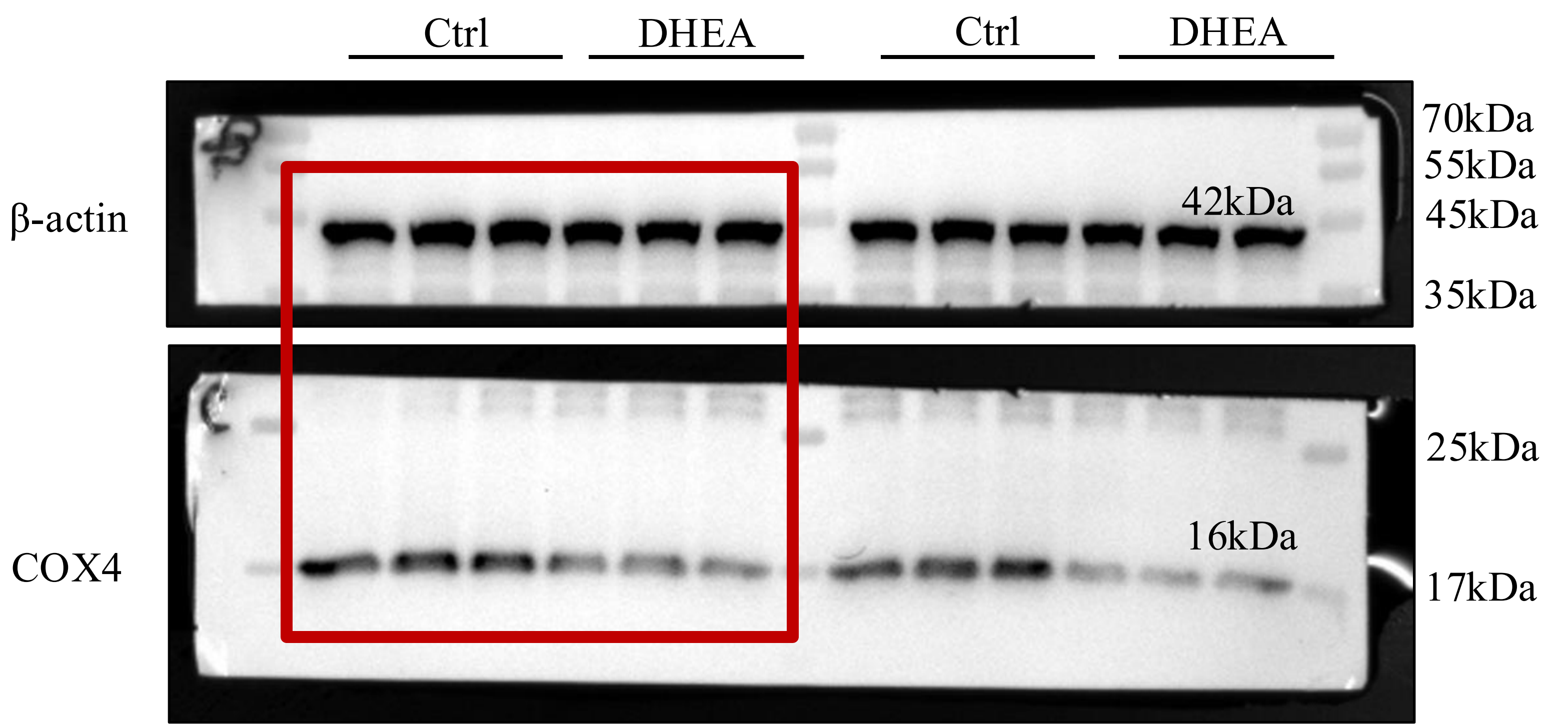

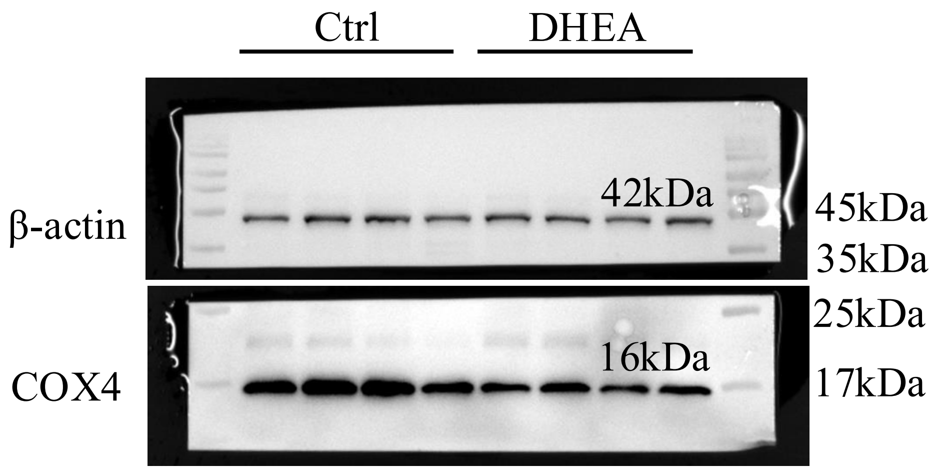

The red boxes show the representative bands exhibited in the text.

Green stars* mark bands resulting from nonspecific hybridization.

Fig. 4F

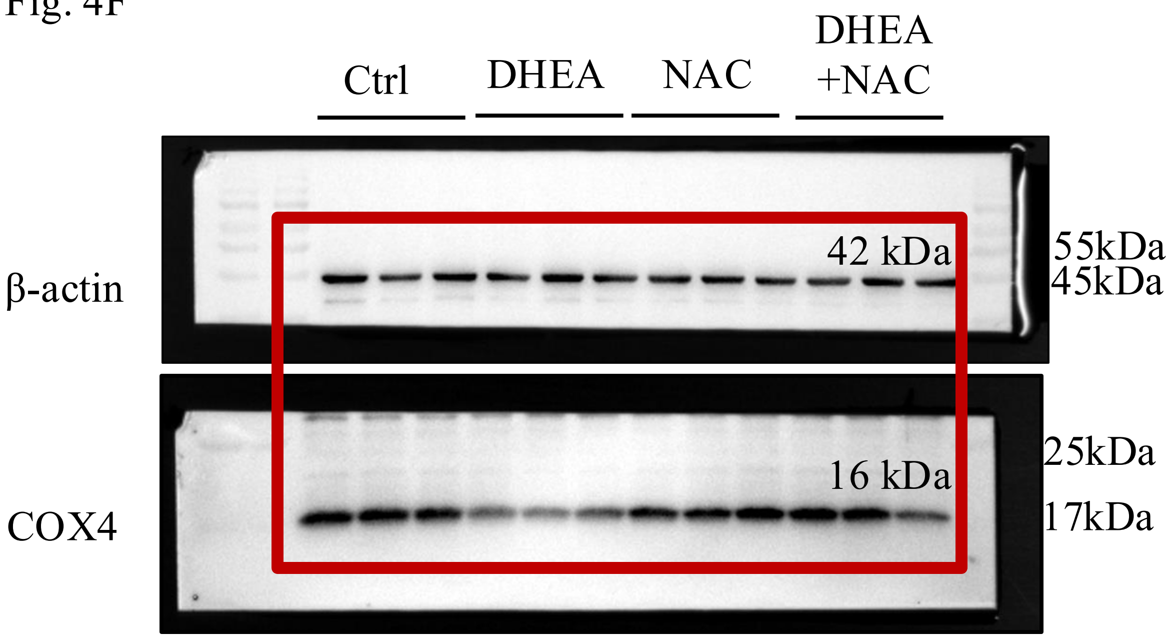

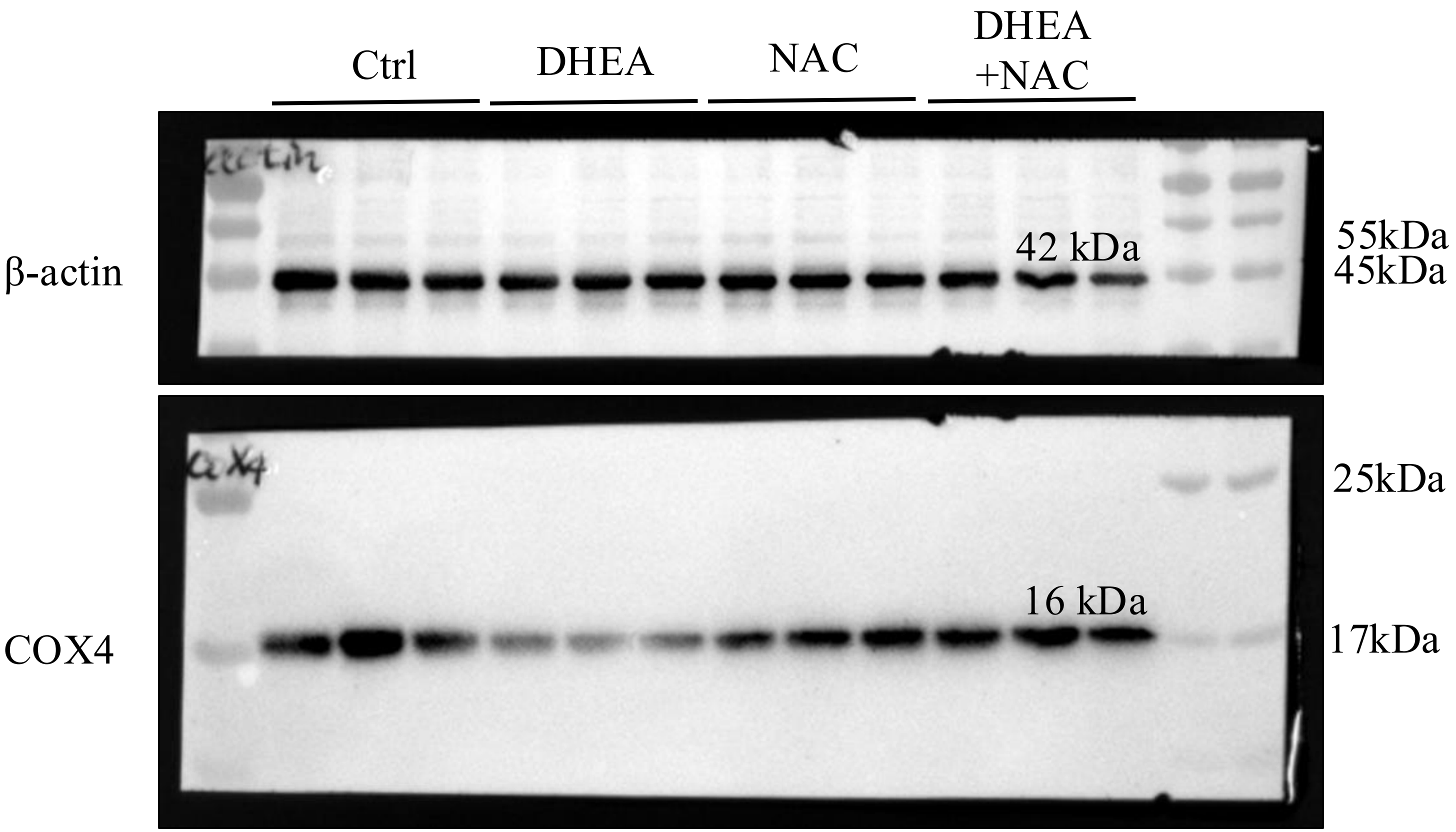

The red boxes show the representative bands exhibited in the text.

Green stars* mark bands resulting from nonspecific hybridization.

Sup Fig. S4A

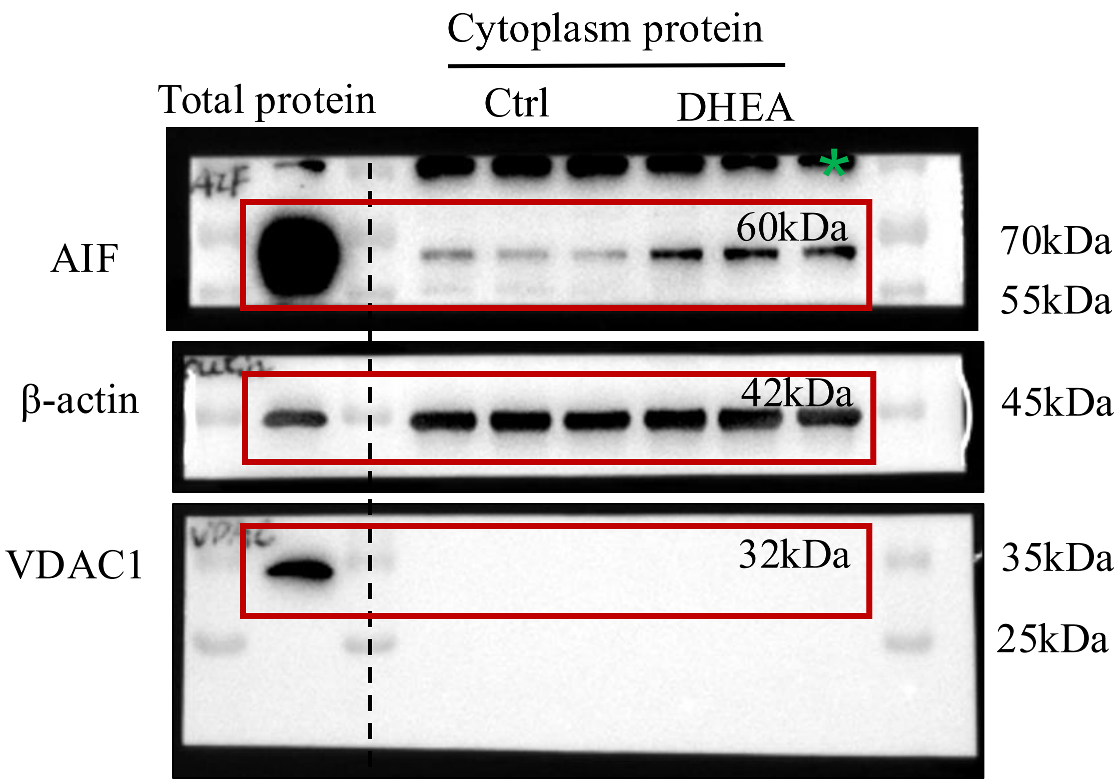

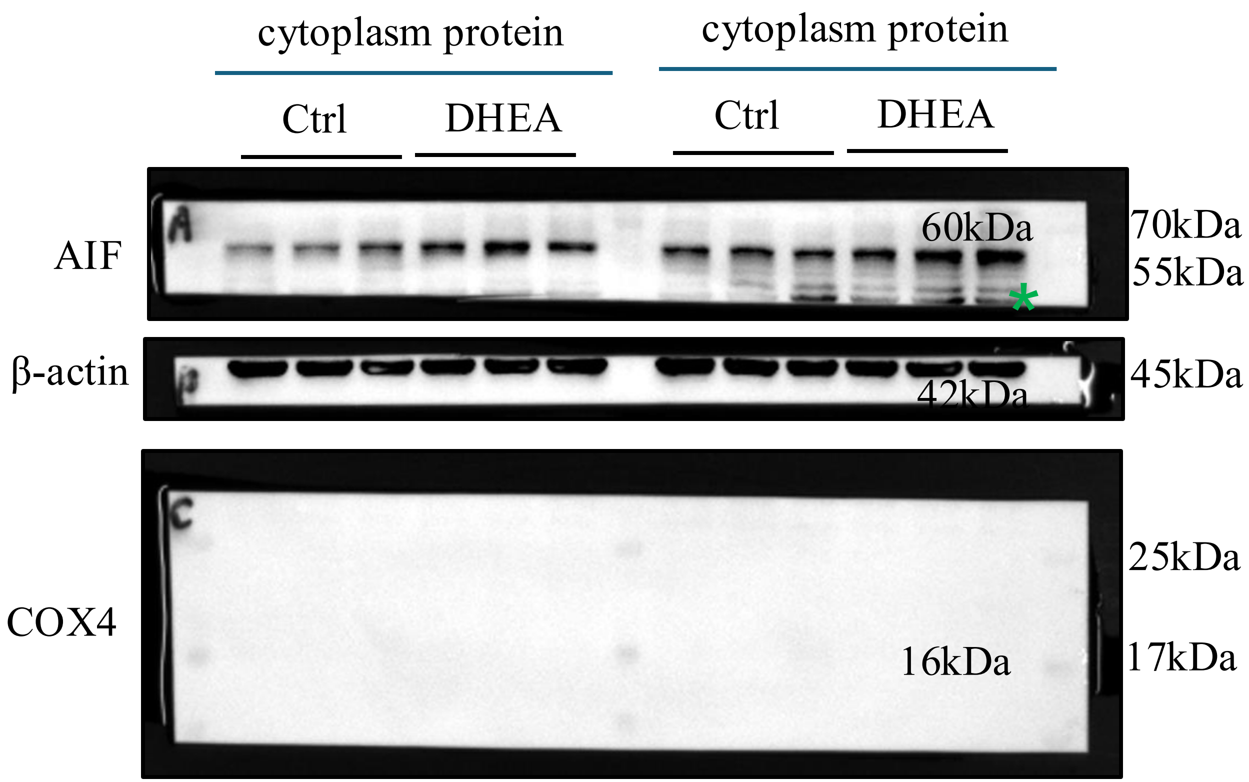

The red boxes show the representative bands exhibited in the text.

Green stars* mark bands resulting from nonspecific hybridization.

Sup Fig. S4C- part 1

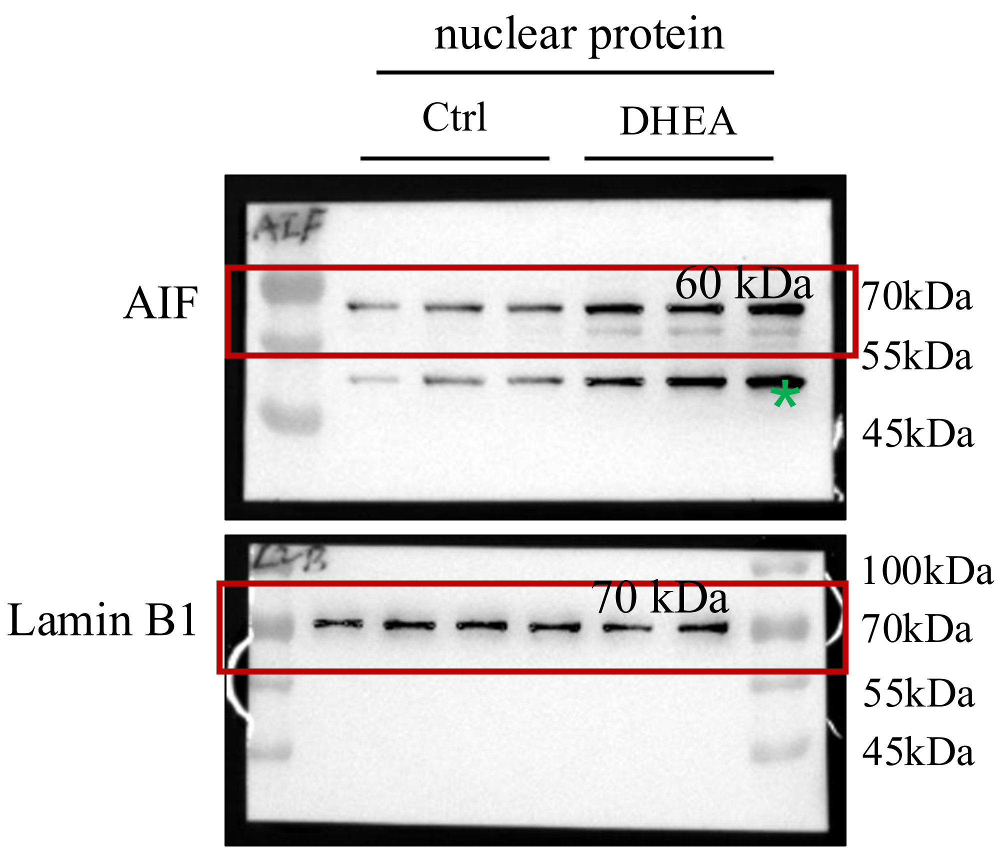

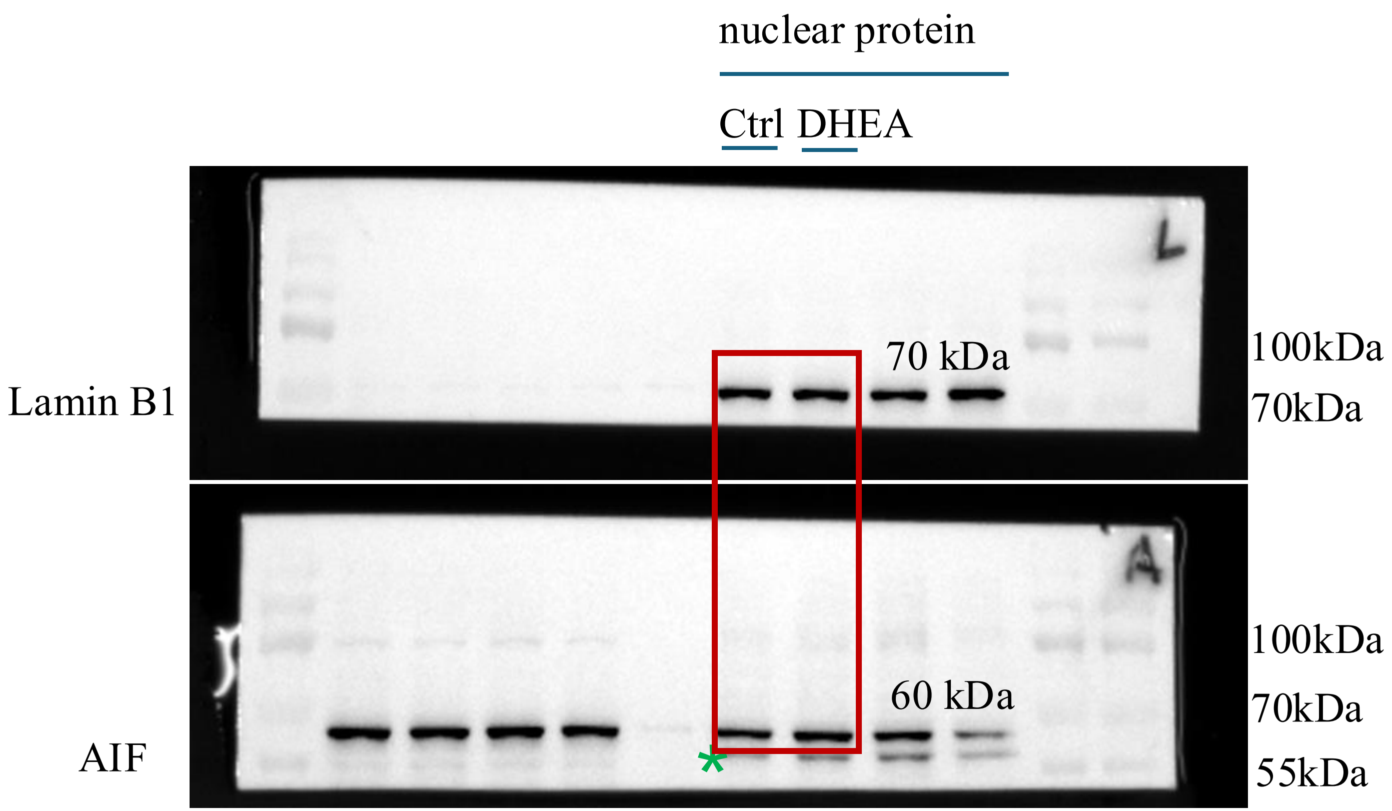

The red boxes show the representative bands exhibited in the text.

Green stars* mark bands resulting from nonspecific hybridization.

Sup Fig. S4C- part 2

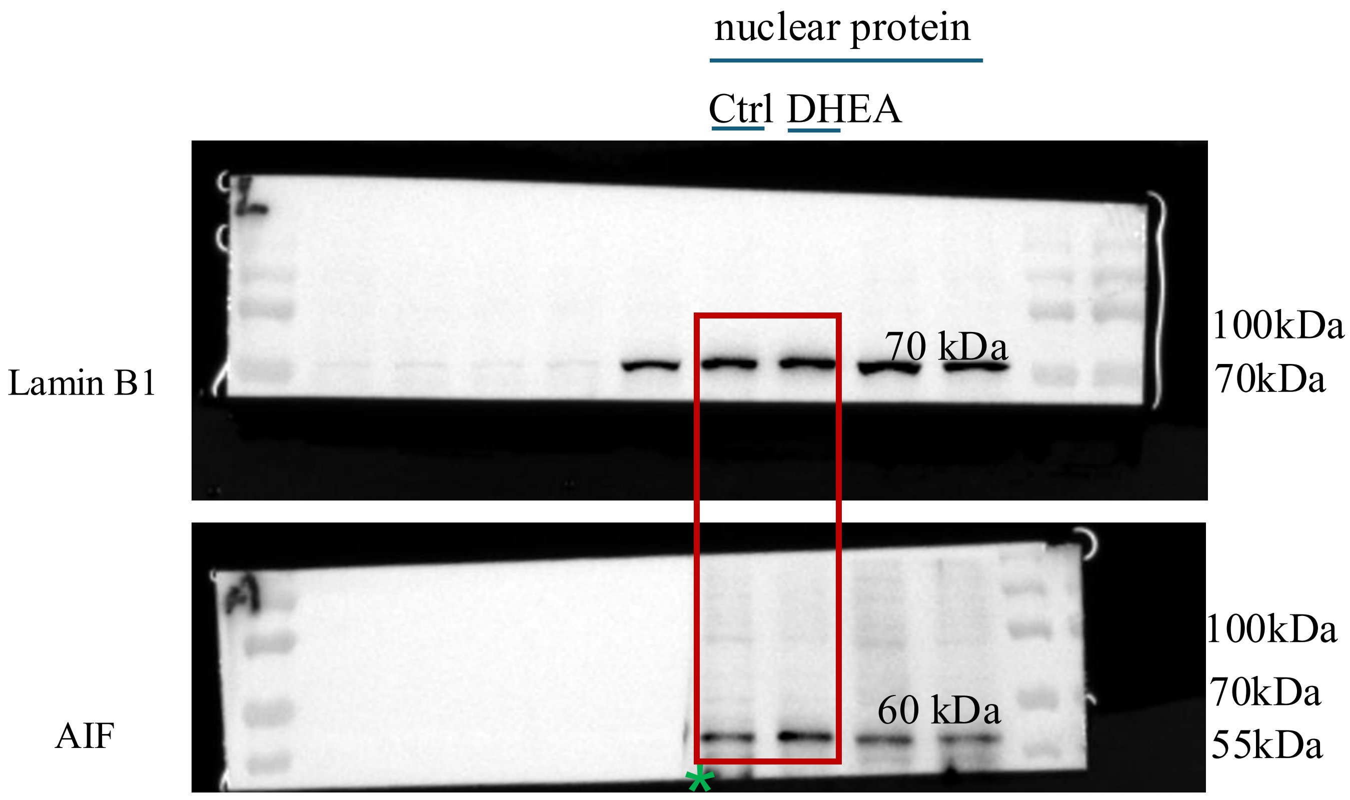

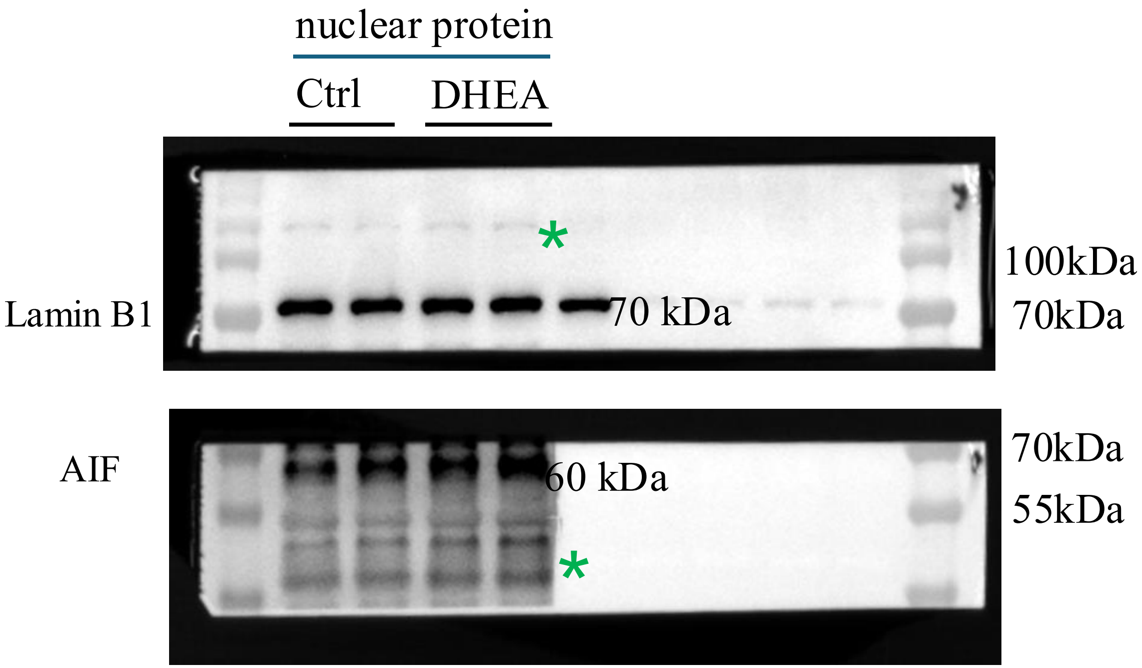

Green stars* mark bands resulting from nonspecific hybridization.
